# Comparison of Soluble Dietary Fibers Extracted from Ten Traditional Legumes: Physicochemical Properties and Biological Functions

**DOI:** 10.3390/foods12122352

**Published:** 2023-06-12

**Authors:** Dingtao Wu, Jiajia Wan, Wenxing Li, Jie Li, Wang Guo, Xiaoqin Zheng, Ren-You Gan, Yichen Hu, Liang Zou

**Affiliations:** 1 Key Laboratory of Coarse Cereal Processing (Ministry of Agriculture and Rural Affairs), Sichuan Engineering & Technology Research Center of Coarse Cereal Industralization, School of Food and Biological Engineering, Chengdu University, Chengdu 610106, China; wudingtao@cdu.edu.cn (D.W.);; 2 Institute for Advanced Study, Chengdu University, Chengdu 610106, China; 3 Singapore Institute of Food and Biotechnology Innovation (SIFBI), Agency for Science, Technology and Research (A*STAR), 31 Biopolis Way, Singapore 138669, Singapore

**Keywords:** legume, soluble dietary fiber, extraction, chemical structure, biological activity

## Abstract

Soluble dietary fibers (SDFs) exist as the major bioactive components in legumes, which exhibit various biological functions. To improve the potential applications of legume SDFs as healthy value-added products in the functional food industry, the physicochemical properties and biological functions of SDFs from ten selected traditional legumes, including mung bean, adzuki bean, red bean, red sword bean, black bean, red kidney bean, speckled kidney bean, common bean, white hyacinth bean, and pea, were studied and compared. Results showed that the physicochemical properties of SDFs varied in different species of legumes. All legume SDFs almost consisted of complex polysaccharides, which were rich in pectic-polysaccharides, e.g., homogalacturonan (HG) and rhamnogalacturonan I (RG I) domains. In addition, hemicelluloses, such as arabinoxylan, xyloglucan, and galactomannan, existed in almost all legume SDFs, and a large number of galactomannans existed in SDFs from black beans. Furthermore, all legume SDFs exhibited potential antioxidant, antiglycation, immunostimulatory, and prebiotic effects, and their biological functions differed relative to their chemical structures. The findings can help reveal the physicochemical and biological properties of different legume SDFs, which can also provide some insights into the further development of legume SDFs as functional food ingredients.

## 1. Introduction

Dietary fibers (DFs) are defined as nondigestible carbohydrates plus lignin [1]. Unlike other food components, DFs possess anti-digestive properties and cannot be digested and absorbed in the small intestine [1]. Generally, DFs contain various indigestible polysaccharides and indigestible oligosaccharides, which mainly exist in different agro-products and agro-byproducts, such as legumes, grains, fruits, and vegetables, and can be classified according to their solubility, viscosity, and fermentability [1,2,3]. DFs are considered to be the basic components of a healthy diet, which are associated with many health benefits. A great deal of evidence shows that high dietary fiber intake can significantly reduce all-cause mortality and reduce the risk of cardiovascular disease, colon cancer, diabetes, and obesity [4,5,6,7]. Due to their high safety, low toxicity, and few side effects, DFs derived from agro-products have attracted increasing attention to be exploited as value-added health products in the health food industry.

The annual output of legumes is second only to wheat, rice, corn, and barley, ranking fifth in the world [8]. Indeed, China is one of the major producers of legumes in the world. In addition to soybean, China possesses various traditional legumes, such as red bean, mung bean, adzuki bean, black bean, red sword bean, red kidney bean, speckled kidney bean, common bean, cowpea, chickpea, and white hyacinth bean [9]. These legumes are rich in DFs, polyphenolics, and proteins [9,10,11,12], which exhibit various health-promoting effects, e.g., cardiovascular protective effect, lowering blood pressure, regulating lipid metabolism, and lowering blood sugar [10,13,14,15]. Specifically, soluble dietary fibers (SDFs) and dietary polysaccharides are the major bioactive components in legumes, exhibiting diverse biological functions, such as antioxidant, anti-diabetic, prebiotic, and immunomodulatory activities [10,11,16,17,18,19,20,21]. To date, the chemical and biological properties of dietary fibers and dietary polysaccharides from soybeans have been widely investigated [10]. Nevertheless, few studies have investigated the physicochemical and functional properties of SDFs from these traditional legumes [10]. More importantly, the comparative studies on the chemical and biological properties of SDFs from different traditional legumes are still limited [11,16]. Therefore, it is important and necessary to compare the physicochemical and functional properties of SDFs from various traditional legumes under the same extraction condition, which can provide some insights into the further development of legume SDFs as functional food ingredients.

To better understand the role that SDFs play in the health benefits of traditional legumes, and to further improve the potential applications of legume SDFs as healthy value-added products in the functional food industry, the structural characteristics and biological functions of SDFs from ten selected traditional legumes were revealed and compared. Firstly, SDFs were extracted from ten selected traditional legumes, including mung bean, adzuki bean, red bean, red sword bean, black bean, red kidney bean, speckled kidney bean, common bean, white hyacinth bean, and pea. Afterward, their physicochemical properties (e.g., chemical compositions, molecular weights, crystalline structures, thermal properties, monosaccharide compositions, and glycosidic linkages) and biological functions (e.g., antioxidant effect, antiglycation effect, prebiotic effect, and immunoregulatory effect) were systematically evaluated and compared. The findings from this study can provide a theoretic basis for the development of legume SDFs as value-added functional/health products in the functional food industry.

## 2. Materials and Methods

### 2.1. Materials and Chemicals

**Figure 1:**
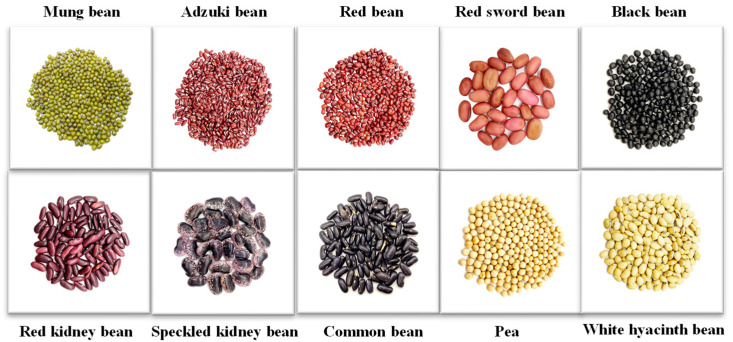
The visual pictures of different traditional legumes.

Ten selected traditional legumes, including mung bean (MMB), adzuki bean (ABB), red bean (RRB), red sword bean (RSB), black bean (BBB), red kidney bean (RKB), speckled kidney bean (SKB), common bean (CCB), white hyacinth bean (WHB), and pea (PPB), were purchased from Ganzhou Kangrui Agricultural Products Co., Ltd. The basic information of ten legumes is shown in Table 1 and Figure 1.

ABTS, DPPH, potassium ferricyanide, carboxymethyl cellulose, trichloroacetic acid, cholestyramine, aminoguanidine (AG), MTT, DMEM medium, thermal-stable α-amylase, pancreatin, sodium nitroprusside, and monosaccharide standards were purchased from Sigma-Aldrich (St. Louis, MO, USA). All other chemicals used in this study were of analytical grade.

### 2.2. Preparation of SDFs from Different Traditional Legumes

SDFs from ten selected traditional legumes were extracted based on a previous method as established by Hu et al. [22]. The dried legume powder (10.0 g) was mixed with 80% methanol (100.0 mL) in an ultrasonic cleaning machine at 480 W (SB-800DTD, Ningbo Scientz Biotechnology Co., Ltd., Ningbo, China) for 30 min to remove most methanol-soluble molecules. Afterward, the pre-treated samples were mixed with distilled water (1:30, *w*/*v*) at 95 °C for two hours to extract crude legume SDFs, which was repeated twice. Then, the extracted supernatants were concentrated using a rotary evaporation (rE-52AA, Yarong, Shanghai, China), and both thermal-stable α-amylase (10 U/mL) and pancreatin (5 U/mL) were sequentially added into the supernatant to remove starch and protein, respectively. After removing starch and protein, both fractional ethanol-precipitation and membrane separation were utilized for further isolation of legume SDFs. The de-starch and de-protein supernatants were precipitated with three volumes of 95% ethanol (*v*/*v*) overnight at 4 °C, and then the precipitates were dissolved in deionized water. The supernatant was then fractionated by using an ultrafiltration device with a molar mass cutoff of 3000 Da (Amicon ultra-15 centrifugal filter, Merck KGaA, Darmstadt, Germany). At last, ten legume SDFs were prepared by lyophilization (−80 °C and 48 h). SDFs from different legumes, including MMB, ABB, RRB, RSB, BBB, RKB, SKB, CCB, WHB, and PPB, were named SDF-MMB, SDF-ABB, SDF-RRB, SDF-RSB, SDF-BBB, SDF-RKB, SDF-SKB, SDF-CCB, SDF-WHB, and SDF-PPB, respectively.

### 2.3. Structural Characterization of SDFs from Different Traditional Legumes

The approximate chemical compositions of SDFs, including total polysaccharides (mg/100 mg), total uronic acids (mg/100 mg), total proteins (mg/100 mg), and total bound polyphenols (mg GAE/g), were analyzed by previous methods [22]; Additionally, molecular weights of SDFs from different traditional legumes were measured by size exclusion chromatography (Wyatt Technology Co., Santa Barbara, CA, USA) with a multi-angle laser scattering detector [23]; Crystalline structures of SDFs from different traditional legumes were analyzed by using a Bruker-AXS D8 Advance X-ray diffractometer (Bruker Optics GmbH & Co. KG, Ettlingen, Germany) [22]; Thermal characteristics of legume SDFs from different traditional legumes were analyzed by using a DSC3500 differential scanning calorimeter (NETZSCH, Rheinstetten, Germany) [22]; Monosaccharide units of legume SDFs from different traditional legumes were analyzed via L-20A HPLC (Shimadzu, Kyoto, Japan) [22]; FT-IR spectral characteristics of legume SDFs from different traditional legumes were analyzed using an Spectrum Two FT-IR spectrometer (PerkinElmer, Waltham, MA, USA) [22]; ^1^H and ^13^C NMR spectral characteristics of legume SDFs from different traditional legumes were analyzed using a Bruker Ascend nuclear magnetic resonance spectroscopy (Bruker, Rheinstetten, Germany) [24].

### 2.4. Evaluation of Biological Functions of SDFs from Different Traditional Legumes

Biological functions of SDFs from different traditional legumes, including antioxidant capacity, antiglycation activity, prebiotic potential, and immunoregulatory activity, were determined by various in vitro models according to our previously reported methodologies [22,25,26]. In detail, antioxidant activities, including ABTS radical scavenging activity, DPPH radical scavenging activity, nitric oxide (NO) radical scavenging ability, and reducing power were assessed using previously described methodologies [25,26]. The antiglycation activity of legume SDFs from different traditional legumes was assessed using a BSA/glucose model as previously described [25]. Additionally, the prebiotic effect of legume SDFs from different traditional legumes was determined using an in vitro batch fermentation model [22]. Furthermore, the immunoregulatory activity of SDFs from different traditional legumes was evaluated using RAW 264.7 macrophages [22], and the effects of different legume SDFs on the production of secretory molecules, e.g., NO, IL-6, and TNF-α, were evaluated.

### 2.5. Statistical Analysis

All results were displayed as means ± standard deviations of triplicate experiments. Origin 9.0 software (OriginLab, Northampton, MA, USA) was applied for the statistical analysis, and statistical significances (*p* < 0.05) were analyzed by a two-tailed Student *t*-test or one-way analysis of variance (ANOVA).

## 3. Results and Discussion

### 3.1. Approximate Chemical Components of SDFs from Ten Selected Traditional Legumes

Table 2 summarizes the approximate chemical components of SDFs from ten selected traditional legumes. The yields of SDFs in different legumes ranged from 8.8 to 31.8 mg/g. The extraction rate of SDF-RKB was the highest (31.8 mg/g), followed by SDF-BBB (25.4 mg/g), SDF-SKB (22.6 mg/g), and SDF-RSB (20.2 mg/g), while the lowest value was found in SDF-PPB (8.8 mg/g) and SDF-RRB (8.8 mg/g). In addition, the contents of total polysaccharides in different SDFs were in the range of 77.58 (SDF-BBB)—93.81 mg/100 mg (SDF-PPB), and the contents of total proteins were in the range of 1.4 (SDF-PPB)—7.88 mg/100 mg (SDF-BBB), suggesting that carbohydrate polymers were the major components in SDFs of different legumes. Similar to a previous study that SDFs extracted from green and yellow peas, kabuli and desi chickpeas, green and red lentils, and navy and pinto beans were mainly composed of a diverse mixture of polysaccharides [27]. Indeed, total uronic acids in different legume SDFs differed from 11.33 to 22.75 mg/100 mg, suggesting that SDFs of different traditional legumes contained pectic-polysaccharides, similar to previous studies that SDFs of legumes are pectin-rich [11,27]. Additionally, the content of uronic acids was the highest in SDF-RKB (22.75 mg/100 mg), while it was the lowest in SDF-ABB (11.33 mg/100 mg) and SDF-CCB (11.79 mg/100 mg). Generally, uronic acids in SDFs are closely associated with their biological functions, e.g., antioxidant capacity, antiglycation effect, and immunomodulatory effect [25,26]. Furthermore, although polyphenolics were removed by methanol extraction, ethanol precipitation, and membrane separation during the sample preparation processes, minor bound phenolic compounds were still found in different legume SDFs, which were in the range of 3.92–27.89 mg GAE/g. This result was comparable to a previous study that dietary polysaccharides extracted from soybean, white kidney bean, red kidney bean, black soybean, field bean, and lentil also contained few polyphenolics in the range of 0.7–1.55% [16]. Indeed, among all legume SDFs, the significantly (*p* < 0.05) higher polyphenolics were found in SDF-RRB (27.18 mg GAE/g) and SDF-BBB (27.89 mg GAE/g). Usually, bound polyphenolics are considered the partial chemical composition of DFs, which can contribute to various biological functions of DFs from natural resources, such as antioxidant, anti-hyperglycemic, and prebiotic effects [28,29,30].

### 3.2. Molecular Weights, Crystalline Characteristics, and Thermal Characteristics of SDFs from Ten Selected Traditional Legumes

**Figure 2:**
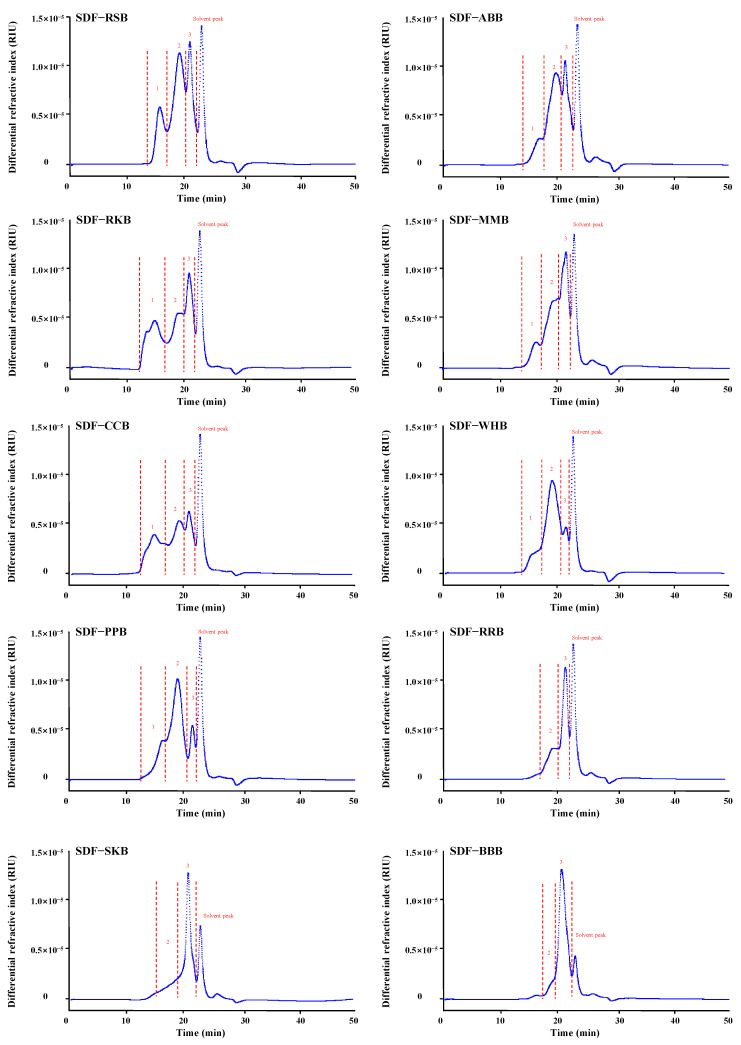
Size exclusion profiles of soluble dietary fibers from different traditional legumes. SDF-RSB, SDF-ABB, SDF-RKB, SDF-MMB, SDF-CCB, SDF-WHB, SDF-PPB, SDF-RRB, SDF-SKB, and SDF-BBB indicate soluble dietary fibers extracted from different legumes, including red sword bean (RSB), adzuki bean (ABB), red kidney bean (RKB), mung bean (MMB), common bean (CCB), white hyacinth bean (WHB), pea (PPB), red bean (RRB), speckled kidney bean (SDF-SKB), and black bean (BBB), respectively. 1, 2, and 3 indicate three different polysaccharide fractions existed in legume SDFs.

Generally, the physical properties (e.g., molecular weight, crystalline property, and thermal property) of SDFs can influence their techno-functional and physiological properties in the food system [22,23]. Therefore, we studied and compared the physical properties of SDFs, e.g., molecular weight, crystalline property, and thermal property. Figure 2 shows the HPSEC chromatograms of ten legume SDFs. As shown in Figure 2, three distinctive fractions, including fraction 1, fraction 2, and fraction 3, were found in SDFs from different traditional legumes, ranging from about 14 to 16 min, 16 to 20 min, and 20 to 22 min, respectively. Specifically, SDF-RSB, SDF-ABB, SDF-RKB, SDF-MMB, SDF-CCB, SDF-WHB, and SDF-PPB had three fractions (fractions 1–3), among which SDF-WHB and SDF-PPB were dominated by fraction 2 (Table 3). Additionally, SDF-RRB, SDF-SKB, and SDF-BBB had two polysaccharide fractions (fractions 2 and 3), among which SDF-BBB was dominated by fraction 3 (Table 3). Furthermore, the detailed molecular weights of different fractions in SDFs and their relative peak areas are summarized in Table 3. As shown in Table 3, molecular weights of fractions 1, 2, and 3 in SDFs ranged from 1.325 × 10^6^ to 2.493 × 10^6^ Da, from 1.059 × 10^5^ to 4.606 × 10^5^ Da, and from 0.801 × 10^4^ to 4.796 × 10^4^ Da, respectively. Overall, these results indicated that molecular weights and contents of different polysaccharide fractions in SDFs varied in different legumes, similar to previous studies [11,16].

**Figure 3:**
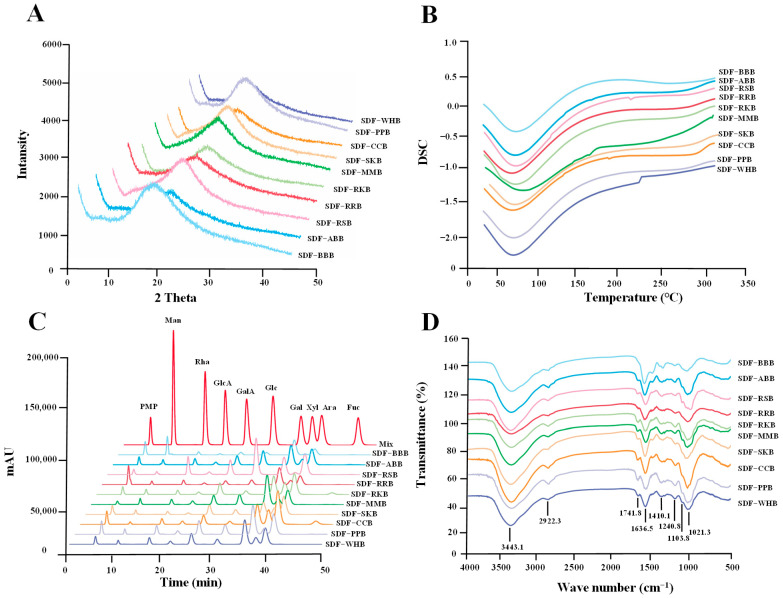
XRD patterns (**A**), DSC thermal spectra (**B**), monosaccharide compositions (**C**), and FT-IR spectra (**D**) of soluble dietary fibers from different traditional legumes.

In addition, the crystal structure of SDFs from different traditional legumes was revealed by XRD analysis. The XRD patterns of ten legume SDFs were recorded from 5° to 50° (Figure 3A). As shown in Figure 3A, the XRD patterns of ten legume SDFs recorded at 2θ of 5–50° were similar, and all SDFs possessed a broad peak and appeared at about 20°, suggesting that all SDFs from different traditional legumes were amorphous polymers [22,31]. Furthermore, the thermal characteristics of SDFs from different traditional legumes were analyzed by DSC analysis. Figure 3B showed that the DSC patterns of ten legume SDFs were similar. Specifically, the transition of the endothermic peak was in the range of 50–100 °C for all legume SDFs and a significant endothermic peak was observed at approximately 70 °C, which may be caused by the evaporation of unbound water [31,32].

The sample codes were the same as in Figure 2.

### 3.3. Monosaccharide Compositions, FT-IR Spectra, and 1D NMR Spectra of SDFs from Ten Selected Traditional Legumes

The chemical structures of SDFs also significantly affect their techno-functional and physiological properties in the food system [22]. Therefore, the monosaccharide analysis, FT-IR analysis, and NMR analysis were performed to compare the primary structures of SDFs extracted from ten selected traditional legumes. Figure 3C shows the HPLC diagrams of monosaccharides derived from different legume SDFs. The findings showed that the HPLC chromatograms of SDF-RKB and SDF-CCB were different from other legume SDFs. In fact, Rha, Man, GlcA, GalA, Glc, Gal, Xyl, and Ara were found in all legume SDFs. However, besides these monosaccharides, Fuc was only found in SDF-RKB and SDF-CCB. Results indicated that monosaccharide units in SDFs varied in different legumes, similar to previous studies [11,16]. In addition, the molar ratios of monosaccharides in SDFs also varied in different traditional legumes (Table 3). Compared with other legume SDFs, only minor Xyl was found in both SDF-RSB and SDF-BBB, and a large amount of Man was found in SDF-BBB. In addition, SDFs from different legumes, including SDF-ABB, SDF-RKB, SDF-MMB, SDF-CCB, SDF-WHB, SDF-PPB, SDF-RRB, and SDF-SKB, were mostly composed of Rha, GalA, Glc, Gal, Xyl, and Ara, despite the fact that their molar ratios differed among different legume species. Usually, the typical monosaccharides of HG and RG I pectic domains contain GalA, Rha, GlcA, Gal, and Ara, and Ara and Gal can also arise from arabinogalactan (AG), suggesting that all legume SDFs contained pectic-polysaccharides, similar to previous studies [11,16,27]. Indeed, the proportion of HG and RG I pectin domains in pectic-polysaccharides can be revealed by the ratio of GalA/Rha (MR1 ratio) [33]. The MR1 ratios of SDF-RSB, SDF-RRB, and SDF-BBB were in the range of 1.11–1.17, suggesting that SDF-RSB, SDF-RRB, and SDF-BBB were rich in RG I pectin domain. However, SDF-RKB and SDF-SKB were rich in HG pectin domain, with the MR1 ratios in the range of 3.57–4.28. Furthermore, Xyl, Glc, and Man are typical monosaccharides of hemicelluloses, e.g., arabinoxylan, glucomannan, galactomannan, and xyloglucan [34,35]. According to the molar ratios of Ara, Xyl, and Glc, arabinoxylan and xyloglucan may exist in almost all SDFs from different traditional legumes, similar to a previous study [27]. Indeed, a large number of galactomannans may also exist in SDF-BBB according to its molar ratios of Gal and Man.

Furthermore, both FT-IR and 1D NMR were applied to analyze the chemical structures of ten legume SDFs. Figure 3D showed that SDFs from ten selected traditional legumes had similar FT-IR spectra, indicating that all legume SDFs had similar functional groups. Specifically, the FT-IR spectra of ten legume SDFs showed typical pectic-polysaccharide absorption bands in the wavelength range of 4000–400 cm^−1^. The strongly tensile characteristic absorption bands of 3443 cm^−1^ and 2922 cm^−1^ were attributed to the stretching vibration of O-H bond and C-H bond [22,25]. The weak absorption band at about 1741 cm^−1^ represented esterified carboxyl groups in ten legume SDFs, and the absorption band at about 1636 cm^−1^ represented free carboxyl groups, further supporting that SDFs contained pectic-polysaccharides [25]. The signal at 1410 cm^−1^ suggested the existence of the symmetrical COO, representing the presence of uronic acid in ten legume SDFs [25]. The existence of pyranose in SDFs and the stretching vibration of C-O-C were, respectively, connected with absorption bands at about 1240 and 1103 cm^−1^ [25]. Moreover, the degree of esterification (DE) of SDFs from different legumes was studied by FT-IR spectroscopy. As shown in Table 2, the DE values of all legume SDFs ranged from 2.67% (SDF-BBB) to 30.43% (SDF-PPB).

**Figure 4:**
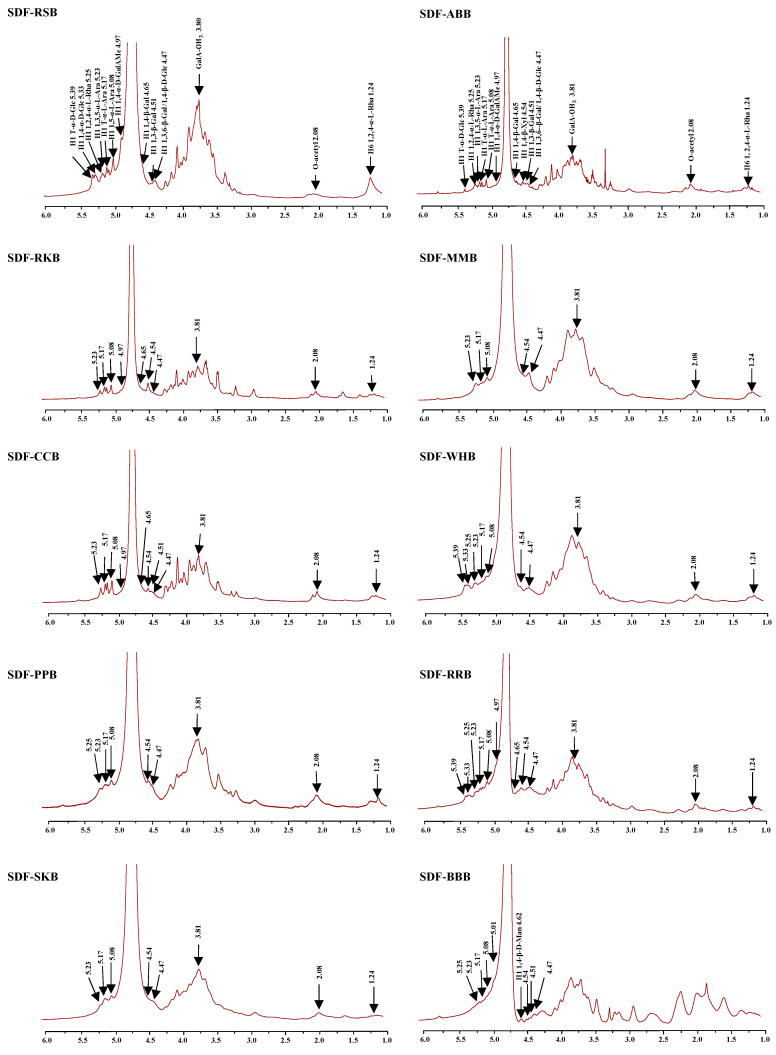
^1^H NMR spectra of soluble dietary fibers from different traditional legumes.

**Figure 5:**
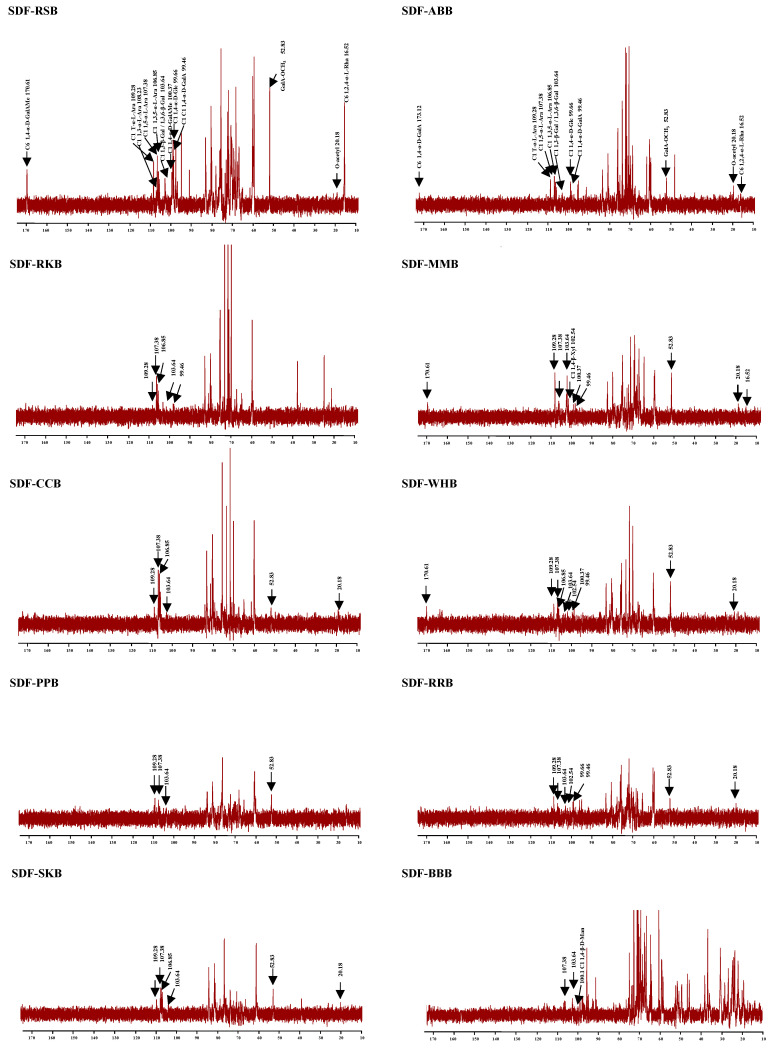
^13^C NMR spectra of soluble dietary fibers from different traditional legumes.

Moreover, Figure 4 and Figure 5 show the ^1^H and ^13^C NMR spectra of ten legume SDFs, respectively. All legume SDFs showed several similar characteristic signals in both ^1^H and ^13^C NMR spectra. In detail, 5.25 ppm (H-1), 1.24 ppm (H-6), and 16.52 ppm (C-6) suggested the presence of 1,2,4-α-L-Rha*p* [36,37]. Additionally, 5.17 ppm (H-1) and 109.28 ppm (C-1) suggested the presence of T-α-L-Ara*f*, 5.08 ppm (H-1) and 107.38 ppm (C-1) were attributed to 1,5-α-L-Ara*f*, 108.23 ppm (C-1) could be attributed to 1,3-α-L-Ara*f*, as well as 5.23 ppm (H-1) and 106.85 ppm (C-1) indicated the existence of 1,3,5-α-L-Ara*f* [36,38]. In addition, 4.97 ppm (H-1), 100.37 ppm (C-1), and 170.61 ppm (C-6) suggested the presence of 1,4-α-D-GalAMe*p*, and 3.81 ppm and 52.83 ppm were attributed to GalA-OCH_3_ [22,25,26]. Additionally, 5.01 ppm (H-1) and 99.46 ppm (C-1) were attributed to 1,4-α-D-GalA*p* [22,26], and 2.08 ppm and 20.18 ppm were attributed to O-acetyl groups [22]. Furthermore, 5.33 ppm (H-1) and 99.66 ppm (C-1) suggested the presence of 1,4-α-D-Glc*p*, and 5.39 ppm (H-1) might be attributed to T-α-D-Glc*p* [36]. Additionally, 4.54 ppm (H-1) and 102.54 ppm (C-1) suggested the existence of 1,4-β-Xyl*p* [36], and 4.62 ppm (H-1) and 100.1 ppm (C-1) suggested the presence of 1,4-β-D-Man*p* [36]. Moreover, 4.65 ppm, 4.51 ppm, and 4.47 ppm might be attributed to the H-1 of 1,4-β-D-Gal*p*, 1,3-β-D-Gal*p*, and 1,3,6-β-D-Gal*p*, respectively [22,36,37]. In addition, 4.47 ppm might be also attributed to the H-1 of 1,4-β-D-Glc*p* [36]. The signal at about 103.6 ppm might be attributed to the C-1 of 1,3-β-D-Gal*p* or 1,4-β-D-Glc*p* or 1,3,6-β-D-Gal*p* [36,38]. Collectively, according to the 1D NMR signals and molar ratios of constituent monosaccharides, all legume SDFs contained pectic-polysaccharides (RG I and HG domains), and hemicelluloses (e.g., arabinoxylan, xyloglucan, and galactomannan) existed in almost all legume SDFs except SDF-RSB. Additionally, a large number of galactomannans existed in SDF-BBB, which are commonly presented in most leguminous seeds [36]. Moreover, resistant starch may exist in SDF-RSB, SDF-WHB, and SDF-RRB due to the signals of 5.33 ppm and 5.39 ppm. Nevertheless, several signals may have overlapped because of the relatively poor resolution of 1D NMR analysis. Thus, further purification and 2D NMR analysis will be conducted in a future study.

The sample codes were the same as in Figure 2.

The sample codes were the same as in Figure 2.

### 3.4. Antioxidant Effects of SDFs from Ten Selected Traditional Legumes

**Figure 6:**
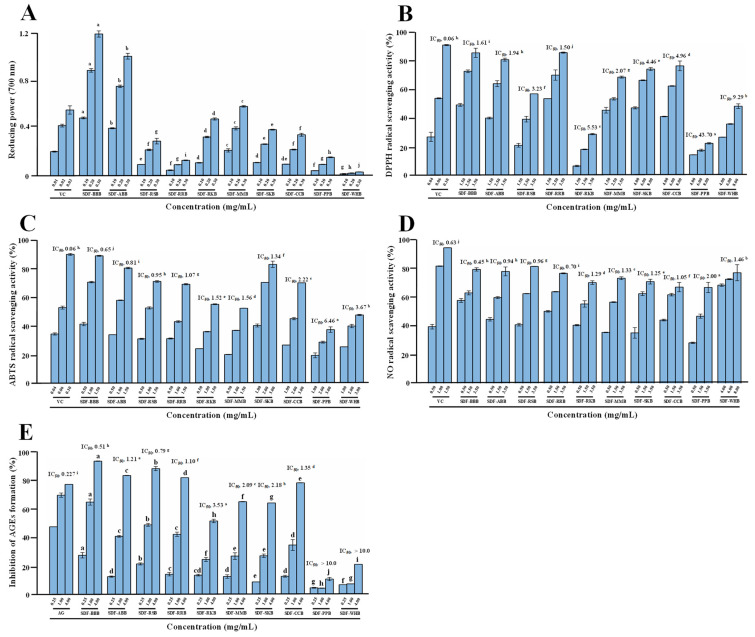
Antioxidant activities and antiglycation activities of soluble dietary fibers from different traditional legumes. (**A**), reducing power; (**B**), DPPH free radical scavenging ability; (**C**), ABTS free radical scavenging ability; (**D**), NO free radical scavenging ability; (**E**), inhibition of AGEs formation. The sample codes were the same as in Figure 2: VC, ascorbic acid; the error bars are standard deviations; significant (*p* < 0.05) differences among different SDFs are shown by data bearing different letters; statistical significances were carried out by ANOVA followed by Duncan’s test.

Several studies have demonstrated that dietary polysaccharides extracted from various legumes possess obvious antioxidant activities [10,19,20,39,40]. However, the comparative studies on the antioxidant activities of SDFs from different traditional legumes under the same extraction and evaluation conditions are still limited. Therefore, the antioxidant activities of ten legume SDFs were assessed by DPPH, ABTS, and NO radical scavenging abilities, as well as reducing power. Figure 6 shows the antioxidant activities of ten legume SDFs. Results indicated that the antioxidant activities of legume SDFs varied in different legume species. As shown in Figure 6A, the values of reducing powers of ten legume SDFs differed from 0.031 to 1.19 at a concentration of 0.3 mg/mL. The strongest reducing power was observed in SDF-BBB among all samples, while SDF-WHB had the weakest reducing power. In addition, as shown in Figure 6B, SDFs from different legumes possessed various scavenging abilities against DPPH free radicals. Among all SDFs, SDF-BBB, SDF-RRB, SDF-ABB, and SDF-MMB showed stronger DPPH free radical scavenging abilities than others, and their IC_50_ values were 1.61, 1.50, 1.94, and 2.07 mg/mL, respectively, similar to previous studies that dietary polysaccharides from mung bean seeds and mung bean skins possessed notable scavenging ability against DPPH free radicals [20,41]. Nevertheless, both SDF-WHB and SDF-PPB possessed poorer DPPH free radical scavenging abilities than other SDFs. Figure 6C shows that SDFs from different legumes also possessed various scavenging abilities against ABTS free radicals, and their IC_50_ values ranged from 0.65 to 6.46 mg/mL. Among all SDFs, SDF-BBB, SDF-ABB, SDF-RSB, and SDF-RRB showed stronger ABTS free radical scavenging ability than others, and their IC_50_ values were 0.65, 0.81, 0.95, and 1.07 mg/mL, respectively. While SDF-WHB and SDF-PPB also had lower scavenging abilities against ABTS radicals, similar to the results of DPPH free radical scavenging ability. Furthermore, Figure 6D shows that SDFs from different legumes also possessed various scavenging abilities against NO free radicals, and their IC_50_ values ranged from 0.45 to 2.00 mg/mL. Compared with VC (IC_50_ = 0.63 mg/mL), SDF-BBB showed higher scavenging activity against NO free radicals, while other SDFs showed moderate scavenging activities against NO free radicals. Additionally, SDF-BBB, SDF-ABB, SDF-RSB, and SDF-RRB also exhibited stronger scavenging abilities against NO free radicals than other SDFs. Overall, the findings indicated that legume SDFs, e.g., SDF-BBB, SDF-ABB, and SDF-RRB, could be developed as potential antioxidants in the food system.

In this study, SDFs extracted from different traditional legumes showed different antioxidant capacities in vitro, which might be closely associated with their physicochemical properties. In fact, many investigations have revealed that the strong antioxidant activity of dietary polysaccharides and dietary fibers is always correlated with low molecular weight, high uronic acid content, and high polyphenolic concentration [22,25,30,42]. Therefore, the combined effect of low molar mass, high content of bound polyphenolic, and high content of uronic acid could contribute to the strong antioxidant activities of SDF-BBB, SDF-ABB, and SDF-RRB. However, to elucidate the detailed relationship between their structures and antioxidant activities, further purification, structural characterization, and in vivo antioxidant activity assay are required.

### 3.5. Antiglycation Activities of SDFs from Different Traditional Legumes

Advanced glycation end products (AGEs) are usually formed by complex amino carbonyl reactions, which can result in an oxidative stress response [43]. Natural antioxidants are beneficial to reducing AGEs, which can ameliorate the oxidative stress response [43]. Studies have demonstrated that legume SDFs possess notable antioxidant capacities. Therefore, this study further studied and compared the antiglycation activities of SDFs from different traditional legumes. The antiglycation activities of ten legume SDFs are shown in Figure 6E, which exhibited potential antiglycation activities in a dose-dependent manner. Specifically, SDF-BBB, SDF-RSB, SDF-RRB, SDF-ABB, and SDF-CCB showed excellent antiglycation activity, and the strongest inhibitory effect against the formation of AGEs was observed in SDF-BBB, with the IC_50_ value of 0.51 mg/mL. Even at 4.00 mg/mL, the antiglycation activity of SDF-BBB (inhibitory rate of 97.97%) was obviously higher than that of the positive control (inhibitory rate of 80.78%), while SDF-PBB and SDF-WHB had lower inhibitory effects on the formation of AGEs than other SDFs. This result was similar to that of the antioxidant activities of different legume SDFs. In fact, the antioxidant activity of pectic-polysaccharides can contribute to inhibiting the formation of AGEs caused by complex amino carbonyl reactions [43]. Therefore, the antiglycation activity of different legume SDFs was closely correlated with their antioxidant activities.

### 3.6. Immunoregulatory Activities of SDFs from Different Traditional Legumes

**Figure 7:**
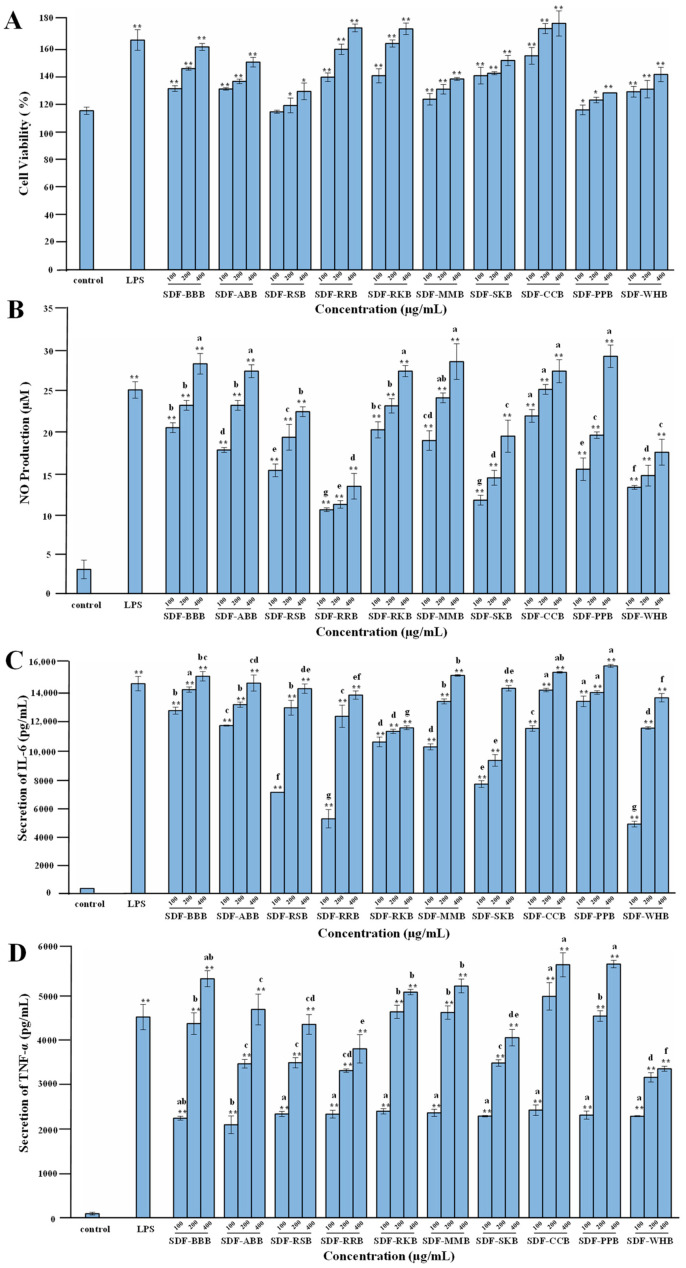
Immunoregulatory effects of soluble dietary fibers from different traditional legumes. (**A**), Cell viability of RAW 264.7 macrophages; (**B**), NO produced from RAW 264.7 macrophages; (**C**), IL-6 secreted from RAW 264.7 macrophages; (**D**), TNF-α secreted from RAW 264.7 macrophages. The sample codes were the same as in Figure 2: Significant differences in cell proliferation and release of NO, IL-6, and TNF-α in LPS and SDFs vs. control are shown by * *p* < 0.05, ** *p* < 0.01. Significant differences (*p* < 0.05) in the release of NO, IL-6, and TNF-α among different SDFs are shown by data bearing different letters.

Several studies have demonstrated that dietary polysaccharides extracted from various legumes, e.g., mung bean, chickpea, and adzuki bean, possess notable immunoregulatory effects in vitro and in vivo [19,21,44], which can significantly increase the release of NO, TNF-α, and IL-6 from RAW 264.7 murine macrophages. However, the comparative studies on the immunoregulatory effects of SDFs from different traditional legumes are limited. Therefore, the immunoregulatory effects of ten legume SDFs were compared. Figure 7 shows the impacts of ten legume SDFs on the cell viability of RAW 264.7 macrophages and the production of secretory molecules, e.g., NO, IL-6, and TNF-α. All legume SDFs showed no toxic effects on RAW 264.7 macrophages at concentrations ranging from 100 to 400 μg/mL (Figure 7A). In addition, as shown in Figure 7B–D, all legume SDFs markedly promoted the release of secretory molecules (e.g., NO, IL-6 and TNF-α) from RAW 264.7 macrophages in a dose-dependent manner at the concentrations in the range of 100–400 μg/mL, suggesting that SDFs from different traditional legumes exhibited potential immunoregulatory effects. In detail, SDF-CCB, SDF-PPB, SDF-BBB, and SDF-MMB showed notably immunoregulatory activities among all legume SDFs, while SDF-SKB, SDF-RRB, and SDF-WHB showed relatively poor immunoregulatory activities compared with other samples. 

In general, structural properties, e.g., chemical composition, molecular weight, type of glycosidic linkage, and degree of chain branching, have important impacts on the immune functions of dietary polysaccharides [26]. Therefore, the differences found in immunoregulatory effects of ten legume SDFs might be attributed to their diverse structural properties. Overall, these findings indicated that these legume SDFs possessed potential immunostimulatory effects. Nevertheless, the potential structure–function relationships and mechanism of actions of these legume SDFs require further investigation.

### 3.7. Prebiotic Potential of SDFs from Different Traditional Legumes

Prebiotic is defined as a substrate that is selectively used by host microbes conferring a health benefit [45,46], and SDFs as prebiotics can selectively regulate the gut microbial composition and promote the release of beneficial metabolites, thus bringing various health-promoting effects [1,3]. In fact, SDFs and dietary polysaccharides from various legumes are resistant to digestion and can reach the colon intact to be utilized by gut microbiota [10]. For instance, dietary polysaccharides extracted from red kidney beans can stimulate the growth of probiotic strains, e.g., *Lactobacillus plantarum* and *L. fermenstum*, which can also stimulate the growth of beneficial gut microbiota (e.g., *Bifidobacterium* spp. and *Lactobacillus* spp.) during the in vitro batch fecal fermentation [18]. In addition, dietary polysaccharides extracted from mung bean can also modulate gut microbial composition by increasing Firmicutes, Bacteroidetes, and Clostridium, and increase the release of SCFAs in vivo [17]. However, comparative studies on the prebiotic effects of SDFs from different traditional legumes have seldomly been conducted. Therefore, we studied and compared the prebiotic potential of SDFs from different traditional legumes.

**Figure 8:**
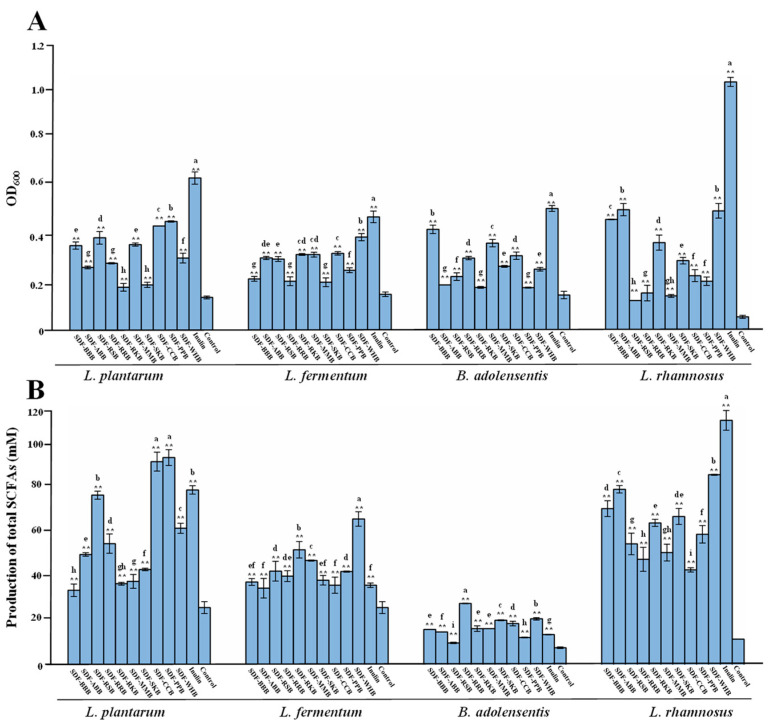
Prebiotic potential of soluble dietary fibers from different traditional legumes. (**A**), the growth of probiotic bacteria; (**B**), the production of short-chain fatty acids from probiotics. The sample codes were the same as in Figure 2: Significant differences (*p* < 0.05) among different SDFs are shown by data bearing different letters. Significant differences in the growth of probiotics and production of total short-chain fatty acids in the positive control and SDFs vs. control are shown by ** *p* < 0.01.

As shown in Figure 8, all legume SDFs tested in the study could markedly simulate the growth of various probiotic bacteria, e.g., *L. plantarum* (CGMCC 1.12974), *L. fermentum* (CGMCC 1.15608), *L. rhamnosus* (ATCC 53103), and *B. adolensentis* (ATCC 15703), similar to that of dietary polysaccharides extracted from red kidney beans [18]. Additionally, the stimulatory effects of all legume SDFs on the growth of various probiotics varied in different legume species, which might be associated with their diverse physicochemical properties. For instance, SDF-BBB could obviously promote the growth of *B. adolensentis*, which might be partially associated with its low molecular weight [22]. Its stimulatory effect on the growth of *B. adolensentis* was close to the positive control (inulin), suggesting that SDF-BBB could be developed as a potential prebiotic for *Bifidobacterium* spp. Moreover, as shown in Figure 8B, all legume SDFs tested in this study could also simulate the release of SCFAs from these probiotics, especially from *L. rhamnosus* and *L. plantarum*, suggesting that all legume SDFs could be partially consumed by these probiotic bacteria to produce beneficial metabolites, e.g., SCFAs. In addition, the stimulatory effects of legume SDFs on the release of SCFAs from probiotic bacteria also varied in different species. The production of SCFAs from *L. plantarum* induced by both SDF-CCB and SDF-PPB was higher than that of others, and the production of SCFAs from *L. fermentum* and *L. rhamnosus* induced by SDF-WHB was also significantly higher than that of others. Overall, these results suggested that legume SDFs could be developed as potential prebiotics for *Bifidobacterium* spp. and *Lactobacillus* spp.

## 4. Conclusions

To better understand the role that SDFs play in the health benefits of traditional legumes and to further improve the potential applications of legume SDFs as value-added health products in the functional food industry, the structural characteristics and biological functions of SDFs from ten selected traditional legumes were revealed and compared. Results revealed that the physicochemical properties of SDFs varied in different species of legumes, e.g., molecular weights of SDF-BBB and SDF-RRB were lower than that of others, and fucose was only observed in SDF-RKB and SDF-CCB. In addition, all legume SDFs were rich in pectic-polysaccharides, e.g., RG I and HG domains. Hemicelluloses, e.g., arabinoxylan, xyloglucan, and galactomannan, existed in almost all legume SDFs, and a large number of galactomannans were found in SDF-BBB. Furthermore, all legume SDFs exhibited antioxidant activity, antiglycation effect, immunostimulatory effect, and prebiotic effect, suggesting that SDFs existed as the major bioactive components in legumes and the dietary consumption of legume SDFs could be a good choice for improving human health. Collectively, the findings in the present study are helpful for a better understanding of the physicochemical and biological properties of SDFs from different traditional legumes, which can provide a theoretic basis for the development of legume SDFs as value-added functional/health products.

## Figures and Tables

**Table 1:** Basic information of ten selected traditional legumes.

Sample Codes	Legumes	Growing Regions
ABB	Adzuki bean (*Vigna angularis*)	Ganzhou, Jiangxi, China
RRB	Red bean (*Vigna angularis*)	Chouyang, Liaoning, China
MMB	Mung bean (*Vigna radiata*)	Baicheng, Jilin, China
RKB	Red kidney bean (*Phaseolus vulgaris*)	Songyuan, Jilin, China
CCB	Common bean (*Phaseolus vulgaris*)	Beijing, China
SKB	Speckled kidney bean (*Phaseolus vulgaris*)	Lijiang, Yunnan, China
BBB	Black bean (*Glycine max*)	Chouyang, Liaoning, China
RSB	Red sword bean (*Canavalia gladiata*)	Shouguang, Shandong, China
WHB	White hyacinth bean (*Lablab purpureus*)	Kunming, Yunnan, China
PPB	Pea (*Pisum sativum*)	Ganzhou, Jiangxi, China

**Table 2:** Chemical compositions of soluble dietary fibers from different traditional legumes.

	SDF-RSB	SDF-ABB	SDF-RKB	SDF-MMB	SDF-CCB	SDF-WHB	SDF-PPB	SDF-RRB	SDF-SKB	SDF-BBB
Total polysaccharides (mg/100 mg)	91.22 ± 0.56 ^ab^	90.49 ± 0.38 ^ab^	90.49 ± 0.15 ^ab^	86.07 ± 2.01 ^c^	86.69 ± 2.34 ^c^	89.36 ± 2.05 ^b^	93.81 ± 5.71 ^a^	85.86 ± 1.46 ^c^	88.04 ± 2.20 ^c^	77.58 ± 4.39 ^d^
Total uronic acids (mg/100 mg)	17.50 ± 0.64 ^b^	11.33 ± 0.38 ^e^	22.75 ± 0.66 ^a^	16.10 ± 0.35 ^c^	11.79 ± 0.49 ^e^	14.07 ± 0.56 ^d^	16.01 ± 0.85 ^c^	16.65 ± 0.58 ^bc^	15.68 ± 0.31 ^c^	13.20 ± 0.59 ^d^
Total phenolics (mg GAE/g)	8.7 ± 0.27 ^f^	23.32 ± 0.69 ^b^	12.03 ± 0.0.50 ^e^	15.77 ± 0.77 ^d^	7.13 ± 0.04 ^g^	6.51 ± 0.33 ^g^	3.92 ± 0.15 ^h^	27.18 ± 0.26 ^a^	18.81 ± 0.34 ^c^	27.89 ± 1.23 ^a^
Total proteins (mg/100 mg)	3.12 ± 0.07 ^d^	2.02 ± 0.25 ^ef^	2.42 ± 0.13 ^e^	4.51 ± 0.46 ^c^	2.43 ± 0.08 ^e^	1.83 ± 0.17 ^f^	0.14 ± 0.02 ^g^	4.16 ± 0.06 ^c^	6.03 ± 0.40 ^b^	7.88 ± 0.50 ^a^
Degree of methylation (%)	20.27 ± 0.41 ^d^	11.17 ± 0.41 ^g^	28.71 ± 0.21 ^b^	20.04 ± 0.46 ^d^	19.47 ± 0.31 ^e^	22.30 ± 0.32 ^c^	30.43 ± 0.59 ^a^	10.41 ± 0.17 ^h^	14.63 ± 0.19 ^f^	2.67 ± 0.43 ^i^

The sample codes were the same as in Table 1. Superscripts differ significantly (*p* < 0.05) among different SDFs extracted from ten traditional beans. Statistical significances were carried out by ANOVA followed by Duncan’s test.

**Table 3:** Molecular weight and composition monosaccharide of soluble dietary fibers from different traditional legumes.

	SDF-RSB	SDF-ABB	SDF-RKB	SDF-MMB	SDF-CCB	SDF-WHB	SDF-PPB	SDF-RRB	SDF-SKB	SDF-BBB
Molecular weight										
Fraction 1 × 10^6^ (Da)	1.325 ± 0.007 ^f^	2.207 ± 0.013 ^b^	2.493 ± 0.047 ^a^	1.821 ± 0.012 ^e^	1.856 ± 0.027 ^e^	2.156 ± 0.013 ^c^	2.016 ± 0.017 ^d^	-	-	-
Relative peak areas (%)	19.6	11.8	35.3	11.7	37.4	12.3	18.9	-	-	-
Fraction 2 × 10^5^ (Da)	1.232 ± 0.008 ^g^	1.284 ± 0.010 ^f^	2.744 ± 0.054 ^b^	1.449 ± 0.011 ^e^	1.705 ± 0.034 ^c^	1.119 ± 0.008 ^h^	1.506 ± 0.015 ^d^	1.059 ± 0.014 ^i^	4.606 ± 0.030 ^a^	1.543 ± 0.015 ^d^
Relative peak areas (%)	49.3	53.3	30.9	44.2	37.0	73.1	65.6	33.5	23.1	8.3
Fraction 3 × 10^4^ (Da)	1.346 ± 0.054 ^e^	2.309 ± 0.062 ^c^	4.798 ± 0.211 ^a^	1.618 ± 0.038 ^d^	4.165 ± 0.141 ^b^	2.329 ± 0.073 ^c^	4.932 ± 0.138 ^a^	0.8007 ± 0.054 ^f^	1.736 ± 0.046 ^d^	1.232 ± 0.049 ^e^
Relative peak areas (%)	31.1	34.9	33.8	44.1	25.6	14.6	15.5	66.5	76.9	91.7
Monosaccharide and molar ratio										
Rhamnose	1	1	1	1	1	1	1	1	1	1
Mannose	0.11	0.54	0.77	0.72	0.29	0.35	0.36	0.49	0.92	3.37
Glucuronic acid	0.24	0.59	0.82	0.97	0.66	0.61	0.50	0.60	0.75	0.54
Galacturonic acid	1.11	1.93	3.57	2.36	2.11	2.17	2.06	1.17	4.28	1.14
Glucose	2.80	3.03	1.29	2.67	0.84	1.04	1.52	1.43	2.21	1.11
Galactose	1.16	3.70	5.20	6.72	4.20	4.26	3.85	4.36	4.84	5.46
Xylose	0.17	1.26	4.00	1.64	3.57	1.42	0.95	1.35	3.02	0.42
Arabinose	1.68	3.37	6.77	3.74	8.02	3.26	3.10	2.64	8.08	2.55
Fucose	-	-	1.78	-	2.17	-	-	-	-	-

The sample codes were the same as in Table 1. Superscripts differ significantly (*p* < 0.05) among different SDFs extracted from ten traditional legumes. Statistical significances were carried out by ANOVA followed by Duncan’s test.

## Data Availability

Data are contained within the article.
